# Requirements for the collection of electronic PROMS either “in clinic” or “at home” as part of the PROMs, PREMs and Effectiveness Programme (PPEP) in Wales: a feasibility study using a generic PROM tool

**DOI:** 10.1186/s40814-018-0282-8

**Published:** 2018-07-04

**Authors:** Susan O’Connell, Robert Palmer, Kathleen Withers, Neeleem Saha, Sarah Puntoni, Grace Carolan-Rees, on behalf of the PROMs, PREMs and Effectiveness Programme

**Affiliations:** 1 HOPE Wales, Cedar Healthcare Technology Research Centre, Cardiff and Vale University Health Board, Cardiff Medicentre, Heath Park, Cardiff, CF14 4UJ UK; 2 NHS Wales Informatics Service, Tŷ Glan-yr-Afon, 21 Cowbridge Road East, Cardiff, CF11 9AD UK; 3 The PROMs, PREMs and Effectiveness Programme, Cardiff and Vale University Health Board, Cardiff, CF14 4UJ UK

**Keywords:** Patient-reported outcome measures, PROMs, Electronic PROMs, E-PROMs, Patient experience, Patient involvement

## Abstract

**Background:**

The patient-reported outcome measures (PROMs), patient-reported experience measure (PREMs) and Effectiveness Programme (PPEP) launched with the aim of supporting all National Health Service Wales (NHS Wales) organisations to collect PROMs and PREMs across a range of conditions. The aim is to collect generic and condition-specific PROMs and PREMs electronically from every secondary care patient in Wales to provide a measure that can be used to determine the clinical and cost-effectiveness of treatments and services. This study reports on the experience of the PPEP in developing an electronic platform suitable for large-scale data collection, storage, analysis and reporting and identifies the problems encountered and solutions implemented using a generic PROM survey as an example.

**Methods:**

The generic PROM survey is available in English and Welsh and consists of a consent section and three components: the EQ-5D-5L tool, the Work Productivity and Activity Impairment (WPAI) tool and a number of “about you” questions. The “about you” questions are designed to assess factors which may affect patient health and outcomes such as information on height, weight, smoking history, exercise levels and alcohol consumption. A dedicated PROM database was built, and links between the e-PROM platform and other key clinical databases within NHS Wales were developed.

**Results:**

Pilot testing of the unvalidated sections of the generic electronic PROM found that most of the questions were well understood and easy to answer: however, feedback suggested some improvements and changes were required, specifically around questions relating to alcohol and exercise.

Electronic PROM collection has been initiated in six of the seven health boards in Wales and at-home collection initiated in three health boards. More than 9300 patients have completed a PROM survey. Early results from one Health Board show that patients took approximately 10 min to complete the questionnaire with most patients answering an average of 94.7% of the questions.

**Conclusions:**

Successful implementation of a PROM collection programme is dependent on a number of factors including close collaboration with clinicians, analysts, IT specialists and patients to ensure that any electronic system of PROM collection is fit for purpose and user friendly both for patients and clinicians.

## Background

In 2008, the Bevan Commission, comprising of a small group of independent experts, was set up to advise the Welsh Minister for Health and Social Services regarding health improvements in Wales. In 2013, the group published Simply Prudent Healthcare [1], a discussion paper on achieving better care and value for money in the Welsh National Health Service (NHS). Prudent Healthcare aims to reduce the historical focus on the volume of activity and the number of medical procedures undertaken, instead placing greater value on patient outcomes.

The principles of Prudent Healthcare support co-production, whereby service users contribute to service provision. The Health Foundation (2010) suggests this model allows for the individualisation of services, dependent on the development of a relationship between the health-care provider and the patient where information and decision making is shared [2]. Furthermore, Prudent Healthcare works with the aim of doing the minimum needed to achieve the greatest patient benefit. This aspiration relies on the availability of evidence of the safety and efficacy of interventions to support decision making.

These aims of Prudent Healthcare can be supported by the use of patient-reported outcome measures (PROMs) and patient-reported experience measures (PREMs) as a means of engaging patients and gathering data to inform future practice.

Increasingly, it is being recognised that patients in Wales have a part to play in improving the quality of the service they receive by participating in the design and delivery of the service themselves—a process known as co-production. Co-production sees patients and clinical staff as equal partners in the planning and delivery of their care to ensure the care a patient receives is appropriate for their needs, beliefs and circumstances. Co-production is based on the sharing of information and on shared decision making between service users and providers and as a result can facilitate greater participation and changes to the traditional balance of power between patients and health-care providers [3]. PROMs and PREMs provide an opportunity for patients to provide feedback from their perspective and are increasingly being used to facilitate co-production in healthcare. PREMs provide insight into the patients’ experience of care providing information on how patients perceived the process of care including factors such as timeliness, cleanliness, staff friendliness and professionalism and dignity. PROMs can help health-care providers to understand the quality of care a patient receives in areas such as effectiveness of treatments leading to improved symptoms and functioning or quality of life and treatment safety (complications or mortality). However, PROMs and PREMs can be extremely helpful at different organisational levels. PROMs and PREMs are a key element of the growing value-based care agenda. NHS England has been collecting orthopaedic PROMs and PREMs since 2009 with individual hospitals using the data collected to inform their processes. The Health and Social Care Information Centre (HSCIC) has produced a benefit case study describing how stakeholders have or are aiming to use the PROM statistical outputs, in order to contribute to improvements in quality of care [4]. The report showed that PROM data are being used in a number of different ways; for example, Northumbria NHS Trust used PROM data to inform their decision to switch implant brands and move away from patella resurfacing for patients undergoing knee surgery. In addition, PROMs allow the calculation of changes in health-related quality of life, and by combining this with cost data, PROMs can be used to estimate the cost-effectiveness of treatments and services. CircleBath, a private provider treating both private and NHS patients, used PROMs to inform the decision to standardise implant brands for hip and knee replacements and as a result has been able to improve procurement rates and reduce costs by bulk ordering [4].

In 2002, the Health Improvement and Patient Outcome (HIPO) project was set up in Cardiff and Vale University Health Board (C&V UHB) Wales. The HIPO project aimed to monitor and improve care using patient surveys to measure quality of life and patient satisfaction. The HIPO project was completed in 2009 having collected more than 96,000 PROMs in a paper format including EQ-5D and Short Form (SF) 36 during its course. The current PPEP is building on the work of the HIPO project, expanding to collect PROMs and PREMs on a national level and moving away from the paper collection of the data by developing a dedicated electronic PROM and PREM data system.

Supported by a number of organisations including the Planned Care Programme Board at Welsh Government and all Welsh Health Boards and Trusts, a proposal to develop a national electronic platform to collect PROMs and PREMs across Wales was submitted jointly by Cardiff and Vale Health Board, NHS Wales Informatics Service (NWIS) and Cedar Healthcare Technology Research Centre. This secured funding in late 2015 via an Efficiency Through Technology Fund (ETTF) grant. This will allow the electronic collection of PROMs and PREMs using either an at-home solution or an in-clinic solution. This will facilitate a move away from paper PROMs where possible and enable the large-scale collection of data across a range of conditions with data available for analyses immediately.

As well as collecting patient-reported data, the platform plans to enable linkage with existing clinical data to facilitate analysis of PROM data and other clinical data widely collected in NHS Wales.

Moving PROM collection to an electronic format involves a number of procedures which are often specified by the owners/licence holders of the individual PROM tool of interest. Following an appropriate methodology such as the ISPOR Guidelines [5] will ensure there is equivalence between paper and electronic PROMs. Legislation in Wales means that all services must be available both in English and Welsh language so there are translation and language validation procedures which must be adhered to. Additionally, as collection of PROMs will happen either in a clinic setting or at home, the electronic system needs to be suitable for use in these different settings.

The long-term aim of the programme is to collect generic and condition-specific PROMs and PREMs from every secondary care patient in Wales to provide a measure that can be used to determine the clinical and cost-effectiveness of treatments and services.

The PPEP has agreed a national set of generic PROMs including EQ-5D-5L (including EQ-VAS) and Work Productivity and Activity Impairment (WPAI) tool [6]. In addition demographic and lifestyle information will be collected through a short series of “about you” questions. Several condition-specific tools have also been agreed including tools for a number of orthopaedic conditions (hips, knees, foot and ankle, shoulders, elbows and hands), paediatric tonsillectomy, lung cancer, asthma and cataracts.

As all patients will complete the same generic PROM, this study uses the generic survey as an example to report on the experience of the PPEP in developing and piloting an electronic platform suitable for large-scale data collection, storage and analysis and reporting. Problems encountered and solutions implemented are identified and discussed. The objective of this pilot study was to determine the feasibility of implementation of the electronic PROM/PREM data collection system within NHS Wales in terms of translation, face validity and usability.

## Methods

### PROM and PREM data collection system

Investigation of the current electronic data collection systems in use revealed a wide variation across the NHS in Wales. Commercial systems are available and in use; however, they are often designed for specific conditions and cannot easily be adapted to collect PROMs in all clinical areas. Commercial systems may also have risks associated with data security, and there are ongoing cost implications to using a commercial system. Some Health Boards have attempted to develop their own in-house electronic solutions; however, these have had limited success and are not scalable across Health Boards, clinics or conditions. Paper collection of PROMs is widespread across Wales, but this presents problems with data storage, standardisation, aggregation and analysis. The development of a dedicated electronic PROM system that allows data to be collected in a standardised manner was considered to be the best approach.

Development of the electronic PROM data collection system consisted of two main components: the building of a dedicated e-PROM system and the development of the links between the e-PROM platform and other key clinical systems within NHS Wales.

The NHS Wales e-PROM system therefore is a complete PROM system that manages and stores all PROM data including templates/formats of questionnaires, patient completion data and rules governing scheduling. The system interfaces with the two main patient administration systems used in secondary care in Wales (Welsh Patient Administration System (WPAS) and the Cardiff and Vale Patient Management System (C&V PMS)) to receive triggers for completion, management of schedules and contacting patients, and patient identification. The national PROM/PREM system is also integrated into the electronic record system, the Welsh Care Records System (WCRS), and can be viewed via the Welsh Clinical Portal (WCP) allowing easy access to view completed forms in the clinic environment to support shared decision making, although this link currently only exists for PROMs collected via the at-home solution.

### E-PROM data collection

The electronic version of the PROM survey was constructed by NWIS as a web-form using Orbeon Forms (v2016.1 and above). Collection of PROMs is being carried out using tablet devices in the clinic setting and using patients’ own devices for at-home collection. The web-based form is optimised for both desktop and mobile devices so that it can easily be accessed by patients and clinicians regardless of device used. Data security is paramount, with regular penetration testing. Information is stored according to national standards, and no data is stored online.

User requirements are continuously being identified in collaboration with clinical leads, service managers, information teams and other key stakeholders across all Health Boards. Problems and difficulties are fed back to the developers, and fixes are implemented at regular update points using agile developments and releases. In-clinic implementation is also supported via staff training, support from PPEP staff and troubleshooting.

After the system has been live in a Health Board or clinic for a period of 4–6 weeks, a system evaluation is conducted and lessons learned are recorded.

### Generic PROM tool

The generic PROM consists of a consent section which includes information on who is completing the questionnaire if on behalf of the patient and why, and three main parts: the EQ-5D-5L tool, the Work Productivity and Activity Impairment (WPAI) tool [6] and a series of “about you” questions (Table 1).

**Table 1:** Generic PROM components

Component	Purpose	Description
Consent		Person completing the form can indicate whether they are the patient or completing the PROM on behalf of a patient Person completing the form provides consent for their responses to be used for clinical and/or research purposes
EQ-5D-5L	Validated tool to assess patient health state	5 dimensions • Mobility • Self-care • Usual activities • Pain/discomfort • Anxiety/depression
Work Productivity and Activity Impairment	Validated tool to measure health-related work productivity loss for the employed population	
About You	Additional questions designed to collect data on important factors which may affect patients health and outcomes	Questions included: • Year of birth • Ethnicity • Medically diagnosed co-morbidities • Height • Weight • Waist size • Smoking status • Alcohol consumption • Exercise level • Employment status

The consent was developed with input from information governance and equality colleagues across Wales. Permission to use the EQ-5D-5L tool on an electronic platform was obtained from the EuroQol Research Foundation [7]. The EuroQol Research Foundation carried out the translation of the EQ-5D-5L from English to Welsh in line with their own requirements.

There are no fees or permissions required to use the WPAI tool, and guidance is provided on how to adapt the tool to an electronic platform. As no Welsh translation was available, translation was carried out following ISPOR guidelines [8].

The “about you” section consists of a series of questions designed to collect data on patients’ personal circumstances and the important factors which may affect their health and outcomes such as information on height, weight, smoking history, exercise levels and alcohol consumption. A question enquiring about medically diagnosed co-morbidities such as high blood pressure, diabetes, depression and arthritis is included with permission from the London School of Hygiene and Tropical Medicine.

Additional “about you” questions were developed based on evidence from the literature and previous surveys. These were developed by the research group and developed iteratively following patient and clinician testing and feedback. These questions were subsequently translated by a recognised translation service, with the outputs tested in a Welsh-speaking cohort of patients and clinicians.

Both the English and Welsh language version of the four sections of the generic PROM were adapted for electronic administration by NWIS. Conversion to electronic format was carried out according to ISPOR Guidelines [5] and guidance from EuroQoL Research Foundation for EQ-5D-5L and Reilly Associates for WPAI [6]. Considerations included factors such as how many questions would appear on each screen, how the questions looked to the user and formatting issues such as font type and size, drop down menus, ability to change responses, and changes to wording to reflect electronic completion.

### Validation of the electronic version of the generic PROM

Patient and clinical staff interviews for the testing and validation of the generic PROM were carried out at Ysbyty Gwynedd, Bangor, North Wales, in June 2016.

The pilot testing was carried out during the e-validation visits whereby members of the PPEP team sat with consenting patients while they completed the PROM survey and discussed any problems with the completion. During this process, members of the NWIS team were also available to deal with any technical issues. Ongoing testing was carried out in clinic supported by the clinical teams who logged onto the PROM system using dedicated tablets provided by the PPEP programme.

Following feedback from patients, clinicians and technical staff, some changes were made to improve the content and format of the generic PROM in line with any relevant licence rules.

### PROM collection in clinic versus at home

Most Welsh Health Boards are actively collecting PROMs; however, there is variation in the type of PROM being collected. Patients referred to secondary care in Wales may now be invited to complete generic and/or condition-specific PROMs depending on the stage of roll-out in their individual Health Boards (Table 2).

**Table 2:** Electronic PROM collection roll-out

Health board	Site	System used	Forms used
Betsi Cadwaladr UHB^a^	Ysbyty Gwynedd	In-clinic	Hip + generic Knee + generic Generic only^b^
	Ysbyty Glan Clwyd	In-clinic	Hip + generic Knee + generic Generic only^b^
	Ysbyty Maelor	In-clinic	Hip + generic Knee + generic Generic only^b^
	Central	At-home	Hip + generic Knee + generic
Aneurin Bevan UHB^a^	Nevill Hall	In-clinic	Hip + generic Knee + generic Generic only^b^
	Royal Gwent	In-clinic	Hip + generic Knee + generic Generic only^b^
Cardiff and Vale University Health Board	Across whole UHB	At-home	Generic only^b^ Shoulder + generic
Hywel Dda UHB	Bronglais General Hospital	In-clinic	Hip + generic Knee + generic
	Withybush General Hospital	In-clinic	Hip + generic Knee + generic Lung cancer + generic
	Glangwili General Hospital	In-clinic	Hip + generic Knee + generic
Abertawe Bro Morgannwg UHB	Morriston Hospital	In-clinic	Tonsillectomy only^c^ Lung cancer + generic
Cwm Taf UHB	Across whole UHB	At-home	Hip + generic Knee + generic

^a^Collection currently on hold

^b^Generic collection has been rolled out across the whole health board, and individual condition-specific tools will be added as they are agreed and validated

^c^Generic survey not active yet

**Fig. 1:**
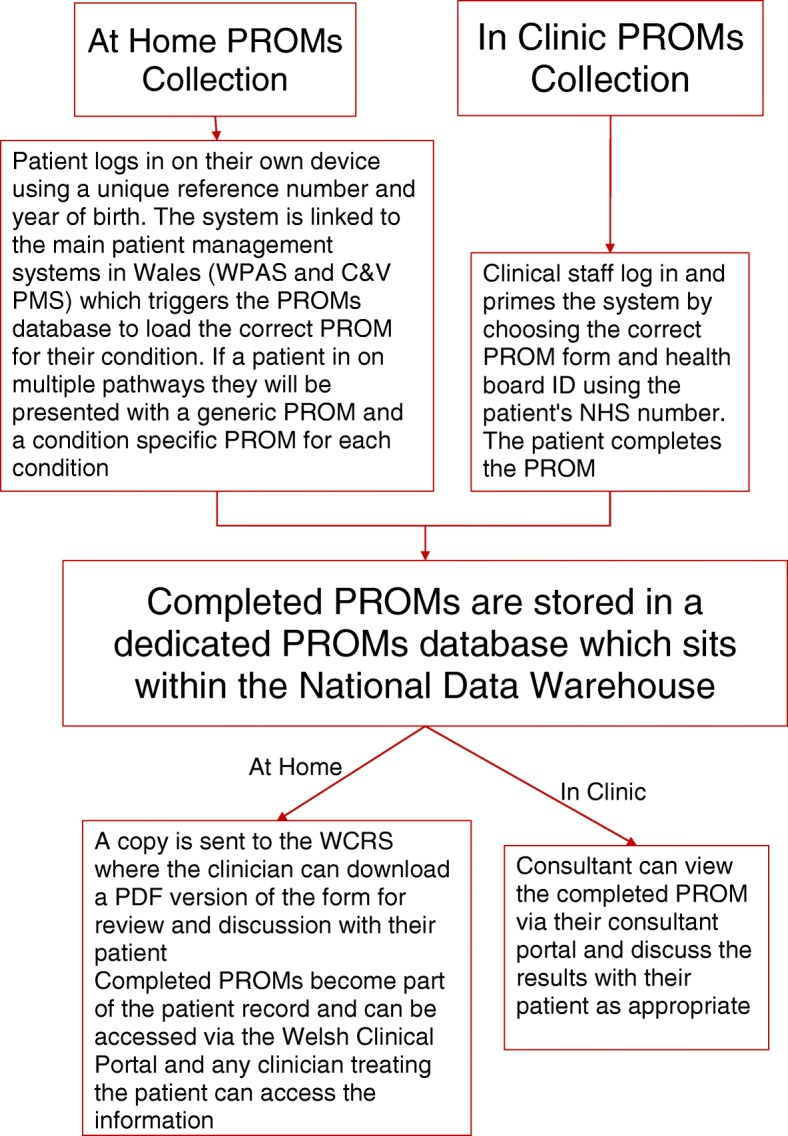
Electronic PROM collection

PROM collection currently occurs either in a clinic setting or at home using electronic solutions which vary slightly. This results in some key differences in the way that data are collected, stored and accessed depending on which solution is used (Fig. 1).

### In clinic PROM data collection solution

PROM collection using the in-clinic solution involves a member of the clinical team logging into the PROM database using a unique identifier (usually the patient’s NHS number) and year of birth. The clinician/staff member selects the correct health board and is then required to select the correct PROM tool from a list for their patient to complete. If a patient requires assistance to complete the PROM, the clinician/staff member can provide this. In-clinic completion should take place prior to a consultation to help inform the patient-clinician decision making process. PROMs collected using the in-clinic method are not added to the global patient record and remain only accessible by consultants using a standalone PROM Clinician’s Portal.

### At-home PROM data collection solution

Collection of PROMs [9] using the at-home solution is facilitated by a link between the two main secondary care Patient Administration Systems (Welsh Patient Administration System (WPAS) and Cardiff and Vale Patient Management System (C&V PMS)) and the PROM system. Welsh PAS and C&V PMS hold patient ID details and record details of patients’ hospital visits, including waiting list management, medical records, inpatient treatment, outpatient appointments and emergency visits. Currently, patients who are referred for treatment in secondary care receive a letter in the post confirming their placement on the waiting list. Prior to the letter being sent, the patient referral to secondary care is seen and triaged by a clinician who ensures that the correct condition pathway is assigned to the patient. Where appropriate, the clinician also assigns the correct PROM to the patient at this point. Welsh PAS and C&V PMS are the systems which facilitate sending the referral acknowledgement letters to patients. Changes made as part of the National PROMs Programme mean that the referral acknowledgement letter now also includes a written link to the PROM website and invites each patient to go online and visit the PROM website. The letter also provides a unique identifier which allows them to access their assigned PROM surveys for completion. The Welsh PAS and C&V PMS also inform the PROM database which condition-specific PROM tool that patient is required to complete alongside the generic survey. Once the patient has completed the PROM, the data are stored in the PROM database; additionally, a notification is sent to the appropriate patient administration system (PAS) so that clinical staff can confirm completion status for their patients. A copy is sent to the Welsh Care Records Service (WCRS). The WCRS is document repository with a reading application which can be accessed by the clinician, and a PDF version of the completed form can be reviewed and discussed with their patient to aid communication and decision making. PROMs completed via the at-home method become part of the global patient record and can be viewed by anyone involved in the care of the individual patient through the Welsh Clinical Portal (WCP). Non-WCP users are still able to access completed PROMs via the PROM Clinician’s Portal.

On a patient-level basis, the PROM responses will aid communications and decision making while collated responses may be used to provide evidence of effectiveness.

## Results

### Translation, face validation and usability testing of the electronic version of the generic PROM

Eighteen patients were approached to take part in cognitive interviews to test the electronic platform. A total of 16 patients agreed to complete an interview and survey while two patients declined to take part. In addition, one member of clinical staff completed the interview and survey.

Validation of the Welsh translation highlighted some minor issues relating to different word meanings. For example, one patient suggested that the Welsh word “cydsyniad” was not a widely recognised word and suggested that “caniatâd” could be used. The differences between these two words are subtle with “cydsyniad” meaning consent and “caniatâd” meaning permission. None of the other patients noted a problem with using cydsyniad.

The clinician suggested that English translations for some of the listed co-morbidities should be included as Welsh-speaking patients may not have encountered biological terms. However, none of the patients interviewed had any difficulties with answering any of these questions suggesting all were able to understand these questions.

Questions on the EQ-5D-5L section were well understood although the electronic layout led to some confusion. EuroQol requirements allow only one question per screen, and some of the questions looked quite similar. This resulted in some patients pressing the “Next” tab again without answering the question as they thought that they had already answered it.

The “sliding scale” on the VAS was a little temperamental, and some patients found this hard to move/slide. Also, if viewing the VAS page on the iPad in portrait view, the “Next” button overlaid the sliding scale and could not easily be pressed. This could be rectified by turning the device to landscape, and the “Next” tab would then stay in the correct place if turned back to portrait and could be easily selected.

The primary issue reported for the WPAI section was that when using the iPad in portrait, the “Finish” button overlaid the final scale. This meant patients had to turn the tablet to landscape to finish the survey.

There were some issues with the “about you” section of the generic PROM which needed to be addressed. Patients generally reported that they would be happy to provide the information requested in the “about you” section but that some of the questions could be clearer or formatted differently to make them easy to answer (see Table 3).

**Table 3:** Electronic validation

Question	Issue	Suggested change
Year of birth	One patient misread this as “Where were you born?” Some patients missed this box or would have if not directed to it. These patients had not noticed the question/box and felt it was not very obvious	To prevent this, consider making this box numerical entry only A larger box and more separation between this and the next question may help with this
Co-morbidities	Patients were asked to select all co-morbidities relevant to them. If patients did not complete this question, it was not clear if it was because they had no co-morbidities or because they skipped the question	Added a “None of the Above” option to the question
Alcohol consumption	1. Patients were asked to enter the number of units consumed. If patients did not complete this question, it was not clear if it was because they did not consume alcohol or because they skipped the question 2. Several patients were not sure what a unit of alcohol was, and as the descriptive sentence is underneath the answer box, they did not see it while considering the question 3. Some patients found it difficult to work out how much they drink “on average” as some weeks they do not drink while other weeks they may have a glass of wine or two for example	1. Added an option for “none” to the question 2. It was suggested that the description should be added at the end of the question, but before the data entry box. Several patients wondered if alternative wording could be used 3. One patient suggested a monthly average to account for variations, while several patients suggested having a range, i.e. approximately how many units of alcohol do you drink on average per week? 0–5 units, 6–10 units, 11–15 units etc.
Smoking	Patients were asked to enter amount they smoke. If patients did not complete this question, it was not clear if it was because they did not smoke or because they skipped the question	Added an option for “none” to the question
Exercise	Several patients exercised regularly (e.g. 4 × 45 min swims, plus several hours of yoga) and found this difficult to add up in minutes	Patients now have the option of a range of values to select from a drop down list, for example, 1–2 and 2–3 h, to make the question more user friendly

The patients interviewed said they would be happy to complete NHS PROM surveys in clinic or at home. Several patients had no or little experience with an iPad but found it relatively easy to use and needed only limited support. The layout and wording was generally easy for them to understand and follow other than those issues detailed above.

All responders said they were happy to share personal information such as alcohol consumption and exercise levels with their clinical teams, with only one patient advising that they would probably be less than 100% honest with these answers.

One patient could not complete the questionnaire on the iPad as she thought the text “was too small” and had forgotten her glasses. Potentially, the text throughout could be increased in size or an explanation regarding the ability to “pinch in” on an iPad to zoom into text could be given by the clinician/clinic staff.

Cardiff and Vale University Health Board was the pilot site for the at-home collection of PROMs. Patient feedback after the at-home collection had been live for a period of 4 weeks highlighted some key issues and led to a number of changes being implemented. For example, patients reported that it was difficult to see where they needed to click to select their questionnaire so changes were made to the homepage to make this more prominent.

Patients reported that the PROM website was not found through search engines. The PROM website is now linked to search engines so that it can easily be found when using search engines.

If a patient has problems with the at-home system, they can e-mail their local PROM contact for help and troubleshooting. An automatic response is triggered which highlights some of the key problems and solutions for the patient to try. If those do not work, a member of the PROM team will investigate further and aim to resolve the issue.

In some cases, patients who have been invited to complete a PROM using the at-home system have reported that they do not have access to the Internet. In these cases, suggestions were made that they could ask a family member or friend for access or they could speak to their clinician about possible access to the in-clinic system. The programme clearly states that completion is not mandatory and responses (or non-response) does not affect individual’s care or waiting times.

### Implementation of electronic PROM collection in Wales

Electronic PROM collection has been initiated in six of the seven Health Boards in Wales since June 2016 with at-home collection initiated in two health boards to date. The generic PROM is currently available in five of the seven Health Boards with at-home collection active in three of these and in-clinic collection active in three Health Boards (Table 2).

There is a continuous process of evaluation of the electronic system. Health Boards can raise any issues with the software which will be investigated to determine whether it is a problem for one or all health boards. Where the problem occurs in one Health Board only, fixes are assessed to ensure they do not impact system function for other Health Boards.

Any fixes or changes to the e-PROM system are made in consideration of both current and future users of the system.

### Early results from the PPEP

Between 21 June 2016 (1st collection date) and 31 December 2017, PROMs have been collected from over 9300 patients.

After data cleaning and formatting, results are available from Cardiff and Vale University Health Board for data collected between 1 January 2017 and 30 September 2017 using the at-home system. A total of 5741 generic PROM forms were collected although some patients completed the form more than once. After removal of duplicates, there were 5366 unique Generic PROMs completed. Where a patient had completed the form more than once, the latest form completed by that patient was used (i.e., the latest PROM form after receiving their letter of referral).

**Fig. 2:**
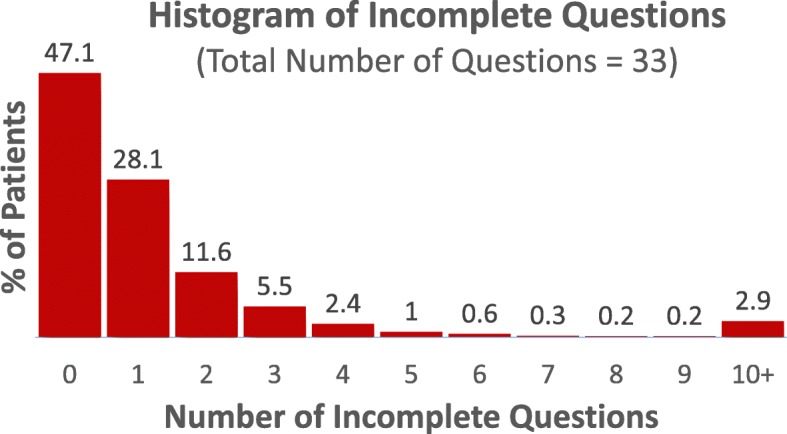
Number of incomplete questions

The generic PROM consists of a total of 33 questions: five introduction questions (including consent), five EQ5D-5L and one EQVAS questions, 16 “About You” questions (i.e., demographics), and six work productivity and activity impairment (WPAI) questions. On average, patients completed 94.5% of the 33 questions, with 47.1% of patients completing all required questions and only 5.5% leaving more than three questions unanswered (Fig. 2).

Furthermore, 94.8% of patients submitted an email address for further electronic communication, while 99.5% of patients completed the PROM in English compared to 0.5% completing in Welsh. 1.6% of the patients who submitted an electronic PROM at-home required assistance in doing so, while on average, patients took 10 min (± 7) to complete the questionnaire once logged-in.

Table 4 below shows the demographics of these patients, which was computed using data collected in the PROM itself.

**Table 4:** Demographic information

Demographic	Group	Number of patients	% of patients
Age	18–29	657	13.9
	30–49	1175	24.8
	50–69	1778	37.6
	70–89	1092	23.1
	90+	27	0.6
Ethnicity	Arab	19	0.4
	Bangladeshi	10	0.2
	Black African	22	0.4
	Black Caribbean	12	0.2
	Chinese	26	0.5
	Indian	33	0.6
	Irish	32	0.6
	Other Asian Background	16	0.3
	Other Ethnic Group	24	0.5
	Other Mixed Ethnic Group	32	0.6
	Other White Background	171	3.3
	Pakistani	27	0.5
	White and Asian	18	0.4
	White and Black African	7	0.1
	White and Black Caribbean	24	0.5
	White British	4667	90.8
BMI	Underweight	109	2.2
	Healthy weight	1847	37.0
	Overweight	1714	34.4
	Obese	1319	26.4
Smoking status	Current smoker	508	9.9
Alcohol use	Over guideline	791	16.5
Exercise level	Does not meet recommended guideline	3606	72.9
Co-morbidity	Heart disease	487	9.5
	High BP	1341	26.0
	Stroke	92	1.8
	Poor circulation	397	7.7
	Lung disease	608	11.8
	Diabetes	484	9.4
	Kidney disease	95	1.8
	Nervous system disease	77	1.5
	Liver disease	48	0.9
	Cancer	241	4.7
	Depression	839	16.3
	Arthritis	1472	28.6
	At least one co-morbidity	3243	63.0
Number of co-morbidities	0	1906	37.0
	1	1570	30.5
	2	900	17.5
	3	458	8.9
	4	195	3.8
	5+	120	2.3
Employment status	In employment	2291	45.4

## Discussion

Paper collection of PROMs does currently happen in a large number of clinics across Wales; however, this is largely driven by individual clinicians working in isolation and impossible to use for large-scale collection analysis or service transformation. Although PROMs are sometimes transcribed to basic electronic systems which could then be linked up to allow aggregate data analysis, this is resource heavy with cost implications. Data collection between different clinics is not standardised, and electronic methods of storage differ greatly making it difficult to link existing systems. There is the additional risk of error when relying on staff to transcribe data to an electronic format for analysis. Paper PROMs may also be subject to different licensing and use rules which may increase costs to the PPEP. Moving to electronic PROM collection also contributes to the strategic commitment towards environmental sustainability by reducing the amount of paper used in NHS Wales.

There may be concerns that electronic data collection does not provide equivalent scores to those obtained using paper and pencil as a result of issues such as differences in how items are presented to a patient or potential difficulties in using electronic systems. Evidence from two separate systematic reviews with meta-analyses [10, 11] supports the idea that electronic collection is equivalent to paper collection of PROMs. One review [10] meta-analysed data from 32 individual studies and reported equivalent scores between computer- and paper-obtained scores. A second review [11] examined evidence from 72 studies concluded that electronic PROM measures can generally be assumed to be equivalent to pen and paper measures and found little evidence that equivalence was compromised by the nature of the condition under investigation. As the PPEP plans to collect not only generic PROMs but also a wide range of condition-specific PROMs, the findings from this review provide reassurance that moving from paper and pen collection to electronic collection will not compromise the data. Collection of PROM data using an electronic platform fits with increasing moves towards telehealth and the monitoring of patient health using electronic and technologic means. This study shows that an efficient and suitable electronic platform can be integrated with existing systems within the health-care system provided appropriate methodology such as the ISPOR Guidelines [5] are employed and adequate testing of the electronic system is conducted.

Moving from paper collection to electronic PROM and PREM collection while preferable is not without its problems. In order for the electronic system to be successful, it needs to be easy to use for both patients and clinicians. The PPEP team work closely with clinicians, health-care professionals and patient representatives to ensure continued engagement and have found them to be enthusiastic and interested in PROM data collection. Clinicians increasingly recognise the benefit of PROMs. Many are already collecting PROMs in some format and see being part of a national programme of data collection as beneficial. Stakeholders appreciate that for the system to work they need to provide feedback, telling the programme what works and what does not. The process of continued evaluation of the electronic system and a regular timetable of updates means that stakeholder feedback is regularly assessed and changes can be implemented quickly and efficiently. There are of course cost implications with setting up a completely new electronic system. It has been estimated that the main costs are associated with set-up and development (clinician time, programme managers, IT infrastructure/time). Once the system is up and running, however, it is anticipated that costs will reduce as it moves into the maintenance stage and changes to the system become less frequent.

Some clinics, while keen to collect PROMs, are not in a position to implement electronic collection. For these clinics, paper collection is a possible solution provided the clinical team are happy to facilitate data collection.

Although the ultimate goal of the PPEP is to move completely to e-PROMs if possible, the value of paper PROM collection is recognised by the PPEP. The possibility of supporting paper collection in a limited capacity where clinics do not have access to the electronic PROM system is being considered.

The electronic PROM data collection system was initially trialled in clinic and found to have a number of potential strengths and weaknesses. These have provided useful learning points and have allowed the system to be updated and improved.

The addition of the “about you” questions is unique to the PPEP, and the hope is that the additional data collected as part of the generic survey will allow in depth analysis of the impact of these lifestyle factors on the health of patients in Wales. Discussion with patients completing the survey in a clinic setting allowed problems with questions to be identified and changes to be implemented. Although the questions were generally well understood and patients were happy to complete them, there were some questions which proved difficult for patients to answer. For example, some of the patients reported not having the information to hand to answer questions relating to height, weight and waist size. Patients did however state that they would be happy to provide this information if facilities were made available in clinic such as tape measures and scales. Allowing patients to provide this information in either imperial (feet/inches for height and stone/pounds for weight) or metric (metres/centimetres for height and kilogrammes for weight) measures provides more choice and allows patients to complete the question without the need for calculations.

The difficulties that patients identified included how best to answer the question relating to alcohol consumption if their consumption varied regularly and some patients were unsure what constituted a unit of alcohol. Changes to the questions, including a more prominent definition of a unit, will hopefully make it easier for patients to provide accurate information.

In-clinic collection of PROMs has some advantages over the at-home collection as the clinical staff will be on hand to answer any questions a patient may have and offer support and assistance when the patient is completing the survey. Additionally, collection of PROMs in a clinic setting offers an alternative for patients who may be keen to complete PROM surveys but who do not have access to suitable technology at home or who may want to seek further information from their clinician before completing a survey.

At-home collection of PROMs was found to have certain advantages over collection in the clinic setting. When PROMs are collected in the clinic setting, a member of the clinical staff is required to log in to the PROM database using the patients’ NHS number as the unique identifier; however, as the in-clinic system is a standalone system and is not linked to any other patient database, there is no way to check the accuracy of the unique identifier. As a result, if a valid NHS number belonging to a different patient is typed in mistakenly, the completed PROM data will be assigned to the wrong patient when it is stored in the PROM database. In comparison, the at-home collection of PROMs is linked to the Welsh PAS and C&V PMS, and when a patient enters the unique identifier provided in their referral acknowledgement letter along with their year of birth, this can be checked and verified against the data held. If the data do not match, the patient will get an error message and will not be allowed to log in as a result ensuring that completed PROM data are always assigned to the correct patient.

Using the in-clinic system, clinic staff are also responsible for selecting the correct PROM tool from a list. Some errors in tool selection have been observed whereas the at-home system is primed by the link with the Welsh PAS and C&V PMS so that when a patient logs in, the correct PROM surveys are already available for them to complete. The advantage with the at-home system means that the PROM data can be added to a patient record and made available to any clinician involved in the care of an individual patient at any time through the Welsh Clinical Portal. This gives the clinician a more complete picture of their patients’ overall health and well-being while providing access to data which may give insight to help develop more appropriate and tailored healthcare, something which cannot currently be done when completing PROMs in clinic.

In-clinic collection relies on clinical staff who are enthusiastic and engaged with the programme and keen on encouraging their patient to complete the survey. Clinical staff need to be willing to explain the programme to their patients and ask them to complete the PROM, and they must be happy to provide support to a patient who might need help completing a survey. There needs to be capacity within the clinics to be able to support the collection with staffing and technical issues the primary concerns. Clinics are busy environments, and collection of PROMs cannot come at the expense of the clinic being able to run efficiently. In addition, the in-clinic collection requires a Wi-Fi connection and access to devices such as tablets or computers which are not available in all locations.

While the optimal solution would be for all PROMs to be collected via the at-home system, the at-home collection of PROMs has been implemented in just three Health Boards to date, with others working towards implementation. Delays have been largely due to:

- Readiness of the local patient management systems
- Development of key features of the PROM/PREM portal and WPAS and C&V PMS systems to support condition-specific collection
- Organisational and clinical engagement

The long-term aim is that all PROM collection will utilise the at-home solution whereby the patient logs in with their unique reference number and the relevant PROM tools are already selected and available. Due to the availability of mobile devices, it is anticipated that patients who have not completed their PROMs at home will be encouraged to do so using their own device to access the at-home PROM system during clinic visits. Collection will continue to take place both at home and in clinic dependent on clinical need and capacity. In-clinic collection, where the clinical staff are responsible for logging the patient in and selecting the correct PROM tool, will remain to support a more flexible collection model such as in a community setting, and as a result, development is on-going and aimed towards resolving the issues around human error when logging patients in and selecting PROM surveys.

PROMs measure health state at a single point in time, and to measure changing health status, multiple PROMS at different time points are needed. Currently, patients will receive an invite to complete a PROM when they are referred to secondary care and patients are asked to provide an e-mail address for future communications. The electronic PROM system has some algorithms inbuilt which will subsequently trigger an e-mail to patients at pre-defined intervals inviting them to complete an up-to-date PROM. Defining triggers for patients on surgical pathways (e.g. orthopaedics) is relatively straightforward as there is a defined intervention (surgery) and invitations to complete a PROM will be sent pre-surgery and post-surgery at 6-monthly intervals. For patients with chronic conditions (e.g. asthma), the situation is more complex and the timing of sending patients with chronic condition PROMs for completion will vary condition by condition. Defining the triggers for conditions takes place in collaboration with the clinical leads in each disease area. An additional layer of complexity is added as patients may be on more than one clinical pathway. A patient on more than one pathway will be eligible to complete both the generic and condition-specific PROM for each pathway they are on. There is a risk that they will be asked to complete generic PROMs too regularly which could lead to patients not completing as they feel it is a burden to them. In order to reduce this risk, the algorithms in the electronic system are being designed to ensure that if a patient has already completed a generic PROM within a defined time period, they will not be asked to complete a new one for another condition. For analysis purposes, this means that within a given time frame, one generic PROM could be linked to a number of condition-specific PROMs for any individual patient.

The PROMs, PREMs and Effectiveness Programme is continuing to grow, with new sites and condition-specific PROM tools being added all the time. Continued engagement of all the Health Boards ensures that the programme constantly improves the systems in place through early identification of problems and implementation of successes making the electronic collection of PROMs on a national scale possible.

## Conclusions

The PROMs, PREMs and Effectiveness Programme is an ambitious programme with a number of hurdles to overcome before the widespread collection of electronic PROM data becomes routine in Wales. This study shows that the successful implementation of a PROM collection programme is possible. Successful implementation is dependent on a number of factors including close collaboration with clinicians, analysts and IT specialists to ensure that any electronic system of PROM collection is fit for purpose and user friendly both for patients and clinicians. The study highlights that for now, future developments need to concentrate on how in-clinic collection can be made more reliable and safe and reduce the risk of human error. This study shows that electronic collection of PROMs on a national scale can be achieved but due to differences in existing IT infrastructure can take time to implement in all location. In the meantime, individual clinicians may continue to collect PROMs using paper-based tools moving to electronic collection as the PPEP is rolled out in their area.

User engagement, involving both patients and clinical staff at different stages of implementation, is crucial to the success of the programme, and focus groups provide essential feedback around the ease of understanding and ease of completion as well as identifying technical problems and glitches with the electronic systems. The successful collection of e-PROMs will depend on a number of factors, one of which is an efficient, easy to use platform.

## Abbreviations

C&V: Cardiff and Vale
e-PROM: Electronic patient-reported outcome measure
ETTF: Efficiency Through Technology Fund
HIPO: Health Improvement and Patient Outcomes
ISPOR: International Society for Pharmacoeconomics and Outcomes Research
PAS: Patient Administration System
PMS: Patient Management System
PPEP: PROMs, PREMs and Effectiveness Programme
PREM: Patient-reported experience measure
PROM: Patient-reported outcome measure
VAS: Visual analogue scale
WCP: Welsh Clinical Portal
WCRS: Welsh Care Records Service
WPAI: Work Productivity and Activity Impairment
WPAS: Welsh Patient Administration System
